# Medical Society of Georgia

**Published:** 1857-04

**Authors:** 


### MEDICAL SOCIETY OF GEORGIA.

The Medical Society of the State of Georgia will convene in the City of Augusta, at 11 o'clock, A. M., on the second Wednesday in April, 1857, and we would urge the medical men of the State to be present upon the occasion. With as much medical talent and learning in proportion to population, as can be found in most of the States of the Union, we are doing almost literally nothing for the advancement of science, simply from the want of a general and thorough organization. We would unite with the Augusta Journal in the remark, that "every physician in the State should consider himself a member of the Society, and we hope, for the interests of the profession, that all will make an effort to attend." With the railroad facilities now found in our State, it will be an easy matter for every quarter to be fully represented in the Society at almost any point, and we hope the Society, in selecting their place for meeting each year, will have regard to every section, and thus excite and keep up the interest of all.

We would, in advance of the meeting, take the liberty of suggesting Atlanta as the next point, and shall confidently expect that the invitation (which will doubtless be presented by the members resident here) will not be disregarded.

